# Effect of Rhythmic Stabilization Technique Versus Conventional Strengthening on Joint Stability, Pain, and Walking Performance in Chronic Ankle Instability

**DOI:** 10.7759/cureus.115133

**Published:** 2026-08-25

**Authors:** Vaibhav V Shinde, Vaishali Jagtap, Aishwarya P Shinde, Maithili V Potdar

**Affiliations:** 1 Department of Physiotherapy, Krishna College of Physiotherapy, Krishna Vishwa Vidyapeeth (Deemed to Be University), Karad, IND

**Keywords:** chronic ankle instability, conventional strengthening, joint stability, pain, rhythmic stabilization technique, walking performance

## Abstract

Background: Chronic ankle instability (CAI) is one of the consequences of repeated sprains of the lateral aspect of an ankle joint, characterized by frequent episodes of the ankle giving way, pain, impaired proprioception, and reduced functional performance. In rehabilitation, traditional strengthening exercises are often performed; however, interventions targeting neuromuscular control and dynamic joint stabilization may provide superior outcomes. Rhythmic stabilization technique (RST), a proprioceptive neuromuscular facilitation technique, promotes co-contraction of agonist and antagonist muscles, thereby enhancing joint stability and proprioceptive function.

Methods: A total of 58 participants aged 18-40 years with a diagnosis of CAI were recruited for this comparative experimental study. Two groups were formed, and participants were randomly assigned to either group: Group A (RST, n = 29) and Group B (conventional strengthening, n = 29). Both groups underwent a structured four-week intervention program. Outcome measures included the Cumberland Ankle Instability Tool (CAIT) for joint stability, Numerical Pain Rating Scale (NPRS) for pain intensity, and Foot and Ankle Ability Measure (FAAM) for walking performance. Assessments were performed pre- and postintervention. Statistical analysis was performed.

Results: Significant improvement was shown by both groups in all outcome measures following the intervention. Group A showed greater improvement in CAIT scores (16.79 ± 2.47 to 28.10 ± 1.21), NPRS scores (7.66 ± 0.81 to 1.66 ± 0.55), and FAAM scores (45.41 ± 3.52 to 74.28 ± 3.76) compared with Group B. Statistically significant differences were observed after the intervention favoring Group A across all outcomes (p < 0.0001).

Conclusion: RST demonstrated superior effectiveness and is considered a valuable evidence-based intervention for the rehabilitation of CAI.

## Introduction

Chronic ankle instability (CAI) is a complex, multifaceted musculoskeletal condition characterized by recurrent incidents of the ankle giving way, persistent feelings of instability, and repeated ankle sprains that persist for more than 12 months following the initial injury [1]. It arises from a combination of mechanical and functional insufficiencies that compromise the structural and neuromuscular integrity of the ankle joint, leading to long-term disability and reduced quality of life. The condition is broadly understood through an updated model that integrates pathomechanical, sensory-perceptual, and motor-behavioral impairments, collectively perpetuating the cycle of instability and reinjury [1].

According to epidemiological studies, a significant percentage of all sports-related injuries are caused by lateral ankle sprains, with a considerable number of individuals progressing to develop CAI following their initial sprain. It is estimated that individuals who sustain a lateral ankle sprain go on to develop chronic symptoms, making CAI a substantial cost on general health with widespread implications for rehabilitation and long-term functional recovery up to 40% [2].

CAI is broadly classified into two subtypes: mechanical instability and functional instability. Important risk factors for the development of CAI include a history of previous lateral ankle sprains, ligamentous hyperlaxity, reduced ankle dorsiflexion range of motion, impaired peroneal muscle reaction time, and deficits in postural control [3]. Mechanical instability refers to objective structural laxity of the ankle ligaments, typically resulting from inadequate healing of the anterior talofibular and calcaneofibular ligaments following acute sprains [4]. Functional instability is referred to as recurrent sensation of the ankle giving way due to impaired proprioception and neuromuscular control, without mechanical laxity.

The mechanism underlying lateral ankle sprains typically involves a combined plantar flexion and inversion force that exceeds the tensile capacity of the lateral ligamentous complex, resulting in partial or complete ligamentous disruption. Accurate diagnosis of CAI requires a thorough clinical assessment that includes a history of recurrent sprains, subjective reports of instability, physical examination for mechanical laxity using tests such as the anterior drawer and talar tilt tests, and functional assessments of balance and neuromuscular performance [5].

Joint stability in the ankle is maintained through a dynamic interplay between passive mechanical restraints provided by ligaments and bone morphology, and active neuromuscular stabilization arising from the surrounding musculature and somatosensory feedback mechanisms. Concurrent somatosensory deficits, including reduced joint position sense and impaired cutaneous receptor function, further diminish the afferent signals required for appropriate motor responses, thereby exacerbating mechanical insufficiency and predisposing the ankle to recurrent injury [6].

Beyond joint instability, CAI exerts a substantial impact on pain perception and walking performance [7]. Individuals with CAI demonstrate significantly altered gait biomechanics, including modified loading strategies, reduced ankle dorsiflexion moments, and asymmetrical ground reaction forces compared with healthy controls.

Given the multidimensional impairments associated with CAI, rehabilitation interventions that target both the mechanical and neuromuscular components of ankle dysfunction are necessary to regain joint stability and functional capacity. The efficacy of exercise therapy was recently examined in a meta-analysis for CAI, which confirmed that targeted exercise interventions significantly improve balance, strength, and functional outcomes, underscoring the central role of structured rehabilitation in managing this condition [8].

Neuromuscular training is a cornerstone of rehabilitation for CAI, with its primary objectives being the restoration of proprioceptive acuity, enhancement of dynamic joint stabilization, and normalization of functional movement patterns. The literature indicates that neuromuscular training programs reduce the frequency of recurrent ankle sprains and improve self-reported function among individuals with CAI [9].

Proprioceptive neuromuscular facilitation (PNF), a specialized approach within neuromuscular training, has gained increasing attention for treating CAI because of its capacity to simultaneously address muscle strength, flexibility, and proprioceptive function through facilitated movement patterns [10].

Conventional strengthening is frequently used as a foundational component of CAI rehabilitation, targeting the peroneals, tibialis anterior, and intrinsic foot muscles to restore the active muscular support of the lateral ankle complex [11]. Despite these benefits, conventional strengthening approaches primarily address isolated muscle performance and may not adequately challenge the dynamic, multiplanar stabilization demands encountered during functional activities, suggesting that supplementary techniques targeting co-contraction and neuromuscular coordination may be necessary [11].

Rhythmic stabilization technique (RST) is a specific PNF technique that involves the application of alternating isometric contractions of agonist and antagonist muscle groups around a joint without any movement, thereby facilitating simultaneous co-contraction, improving joint stability, and enhancing proprioceptive input. In the context of CAI, where neuromuscular deficits and impaired reflexive stabilization are primary contributors to recurrent instability, RST offers a mechanistically sound approach to restoring the neuromuscular competence necessary for dynamic ankle joint protection [10].

Additionally, ultrasound studies have documented measurable morphological changes in the periankle musculature, including alterations in muscle thickness and cross-sectional area, particularly in the peroneal muscle, which serves as a primary dynamic stabilizer [12]. The management of pain in the context of CAI and associated peroneal tendon pathology has also been addressed through electrotherapy modalities, with evidence suggesting the efficacy of ultrasound and interferential therapy in reducing pain and improving quality of life in patients with ankle sprain [13].

The present research has provided benefits of both conventional strengthening and PNF-based approaches individually for CAI rehabilitation; a direct comparative evaluation of RST against conventional strengthening, specifically examining their effects on joint stability, pain, and walking performance, remains conspicuously absent from the literature. Prior studies have assessed neuromuscular training broadly, without isolating the specific contribution of RST as a distinct intervention or its relative superiority over conventional strengthening [9]. The current investigation, which attempts to ascertain whether RST produces better treatment effects than conventional strengthening in individuals with CAI, was motivated by this void in the literature, thereby providing evidence-based guidance for optimizing rehabilitation protocols in this population.

## Materials and methods

Study design

The study used a comparative experimental approach to conduct research into differences in the impacts of RST vs. Conventional Strengthening on joint stability, pain, and walking performance in CAI individuals. The study was conducted at Krishna Vishwa Vidyapeeth, Karad, from November 2025 to May 2026.

Selection of subjects

Participants were randomly allocated into two groups: Group A (RST) and Group B (Conventional Strengthening) to facilitate a rigorous comparative analysis of treatment outcomes between the two intervention protocols. Simple random sampling was used to enroll 58 participants in total for the study.

The formula was used to determine the sample size:

$n = \frac{4 \times (SD)^2}{(\mathrm{Mean} \times \varepsilon)^2}$

where SD is the standard deviation, mean is the population mean, and ε is the allowable margin of error. This calculation yielded a final sample of n = 58 subjects. Participants were selected from the age group of 18-40 years, comprising both male and female participants, who had a confirmed diagnosis of CAI. Two groups were formed: Group A and Group B, with 29 participants in each, to enable a balanced comparative analysis of the two intervention protocols.

Inclusion criteria

To qualify for inclusion in this study, participants had to meet a defined set of eligibility criteria. Eligible individuals were men and women aged 18-40 years. A confirmed diagnosis of CAI was established using a Cumberland Ankle Instability Tool (CAIT) score of 24 or below, which met the CAI criterion. Participants were also required to report a history of repeated ankle sprains occurring within the preceding six months. To clinically verify mechanical instability of the ankle joint, both the anterior drawer test and the talar tilt test had to yield positive results. Finally, enrolment was limited to those who expressed voluntary willingness to take part in the research and signed the informed consent form prior to participation.

Exclusion criteria

Individuals having either of the subsequent conditions were not allowed to participate in the study. People having a history of malignancy or lower limb fractures were not allowed to participate. Those with a recent history of lower limb surgery were similarly excluded to avoid confounding the intervention outcomes. The presence of any neurological disorder affecting lower limb control was also an exclusion criterion, as such conditions could independently influence neuromuscular function and balance. Finally, individuals who were concurrently enrolled in another rehabilitation or sports performance program were excluded.

Selection of outcome measures

Cumberland Ankle Instability Tool

Joint stability was evaluated, and the degree of CAI was measured using CAIT. A score of 24 or lower on this validated nine-item self-report questionnaire, which has a maximum score of 30, indicates the existence of CAI. A CAIT score of 24 or lower was established by Gribble et al. in the International Ankle Consortium's position statement on selection criteria for CAI research as the verified threshold for verifying CAI in controlled research settings [14].

Numerical Pain Rating Scale

Numerical Pain Rating Scale (NPRS) was used to assess pain intensity in all participants. It is a simple, reliable, and sensitive tool in which participants rate their pain on a scale of 0 to 10, with 0 indicating no pain and 10 the worst imaginable pain. Jensen and McFarland demonstrated strong reliability and validity of the NPRS for measuring pain intensity in chronic pain cases, across the intervention period [15].

Foot and Ankle Ability Measure

Using the Activities of Daily Living and Sports subscales, the Foot and Ankle Ability Measure (FAAM) was used to assess walking performance and foot and ankle functional ability; higher scores indicated more functional ability. Using Rasch analysis, Matheny and Clanton verified the validity and reliability of FAAM scores, demonstrating robust measurement characteristics such as item fit and unidimensionality, thereby supporting its use as a reliable outcome measure for people with CAI [16].

Procedure

The study was initiated by identifying participants, followed by systematic screening of all potential participants against predefined inclusion and exclusion criteria. A total of 58 participants were enrolled in the study. Prior to enrolment, study objectives and procedures were clearly explained to each participant, and written informed consent was obtained from all. Following enrolment, demographic data were collected, including age, gender, duration of symptoms, and body mass index. All participants subsequently underwent a preintervention assessment at baseline, which included evaluation of joint stability using the CAIT, pain intensity using the NPRS, and walking performance using the FAAM. Participants were then randomly allocated into two equal groups of 29 each. Group A received the RST, while Group B underwent Conventional Strengthening exercises. Both groups followed a structured four-week intervention protocol.

At the conclusion of the intervention period, a postintervention assessment was conducted using the same outcome measures, CAIT, NPRS, and FAAM, to evaluate changes from baseline. The collected data were subjected to statistical analysis. The findings were then interpreted to derive the results and conclusions of the study.

Treatment protocol

Participants were randomly assigned to two groups after the baseline evaluation and treated according to the four-week intervention protocol outlined below. Each session commenced with a warm-up phase and concluded with a cool-down phase. Progressive overload principles were applied across weeks, with exercise complexity and resistance increased systematically to match participant progress, consistent with the phased progression approach adopted in the reference study. In Group A, RST was performed by participants in progression of exercise repetitions, as given in Table 1.

**Table 1:** Treatment protocol for individuals in Group A (RST) RST: rhythmic stabilization technique; reps: repetitions

Week	Exercises	Repetition
Week 1	Calf raises	3 × 10 reps
	Single-leg standing	3 × 30 seconds hold
	Weight shifts on a balance pad	3 × 10 shifts each direction
	RST in single-leg stance	3 × 30 seconds hold
Week 2	Week 1 exercises	As per Week 1
	Single-leg stance with eyes closed	3 × 20-30 seconds each leg
	Heel to toe walking	3 × 10 steps (both directions)
	Step-ups	3 × 10 reps
	RST in mini squats	3 × 30 seconds
Week 3	Week 2 exercises	As per Week 2
	Target hopping in multiple directions	3 × 10 hops each direction
	Lateral hops	3 × 10 reps
	Side-to-side step overs	3 × 10 reps
	Single-leg balance with ball	3 × 10 reps
	RST with unstable surface	3 × 30 seconds per leg
Week 4	Week 3 exercises	As per Week 3
	Straight-line running	4 × 20-30 m
	Agility ladder drills (forward/backward)	3 × 1 minute
	Cone zig-zag runs	3 × 4 cones
	Single-leg mini jumps	3 × 15 reps
	RST during walking	3 × 30 seconds sets

In Group B, Conventional Strengthening Exercises were performed using the TheraBand in progression, as given in Table 2.

**Table 2:** Treatment protocol for participants in Group B (Conventional Strengthening) reps: repetitions

Week	Exercises	Repetition
Week 1	Yellow TheraBand - ankle dorsiflexion	3 × 10 reps
	Ankle plantarflexion	3 × 10 reps
	Ankle inversion	3 × 10 reps
	Ankle eversion	3 × 10 reps
Week 2	Yellow TheraBand - all Week 1 exercises	3 × 10 reps each
	Single-leg calf raises	3 × 10 reps
	Towel scrunches (intrinsic foot muscles)	3 × 10 reps
Week 3	Red TheraBand - dorsiflexion, plantarflexion, inversion, eversion	3 × 10 reps each
	Double-leg calf raises	3 × 10 reps
	Step-ups on stable surface	3 × 10 reps
	Single-leg balance with resistance band	3 × 10 reps
Week 4	Red TheraBand - all Week 3 exercises	3 × 10 reps each
	Side lunges	3 × 10 reps
	Hopping drills (forward/backward)	3 × 2 rounds
	Lateral band walks	3 × 10 reps each leg
	Advanced functional strengthening (single-leg squats, step-downs)	3 × 10 reps each leg

Statistical analysis

All statistical analyses were performed using IBM Statistical Package for Social Sciences Statistics, version 26.0 (IBM Corp., Armonk, NY). Normality of data distribution was first assessed using the Shapiro-Wilk test, which confirmed that all outcome variables were normally distributed; hence, the assumptions for parametric testing were satisfied. Within-group comparisons were performed using the paired-samples t-test, and between-group comparisons were carried out using the independent-samples t-test. All analyses were carried out at a 95% confidence level (α = 0.05), with results reported as mean ± SD where applicable. A p value below 0.05 was considered statistically significant.

## Results

A total of 58 participants were included in the study, with 29 participants each in the control and experimental groups. The majority of participants were aged 18-25 years (60.3%), while the remaining participants were aged 26-40 years (39.7%). The sample comprised 33 male participants (56.9%) and 25 female participants (43.1%). Regarding symptom duration, 41.4% had ankle sprain symptoms for four to six months, 37.9% for one to three months, and 20.7% for 7-12 months. The distributions of age and symptom duration were nearly identical between the two groups, and the gender distribution showed only slight variation, indicating that the groups were demographically comparable at baseline, as shown in Table 3.

**Table 3:** General characteristics of the study subjects N = 58; Group A (n = 29); Group B (n = 29)

Demographic variables	Parameters	Total participants	Group A	Group B
Age	18-25 years	35	18	17
	26-40 years	23	11	12
Gender	Males	33	15	18
	Females	25	14	11
Duration of ankle sprains	1-3 months	22	11	11
	4-6 months	24	12	12
	7-12 months	12	6	6

Both groups showed statistically significant improvements from pre- to postintervention across all three outcome measures, but Group A demonstrated clearly superior results compared with Group B. Overall, the higher t-values and stronger significance level (p < 0.0001) for Group A across all outcomes are shown in Table 4.

**Table 4:** Comparison of mean scores of outcomes within and between both the groups Within-group pre- to postintervention differences were analyzed using the paired-samples t-test; a p value of <0.05 was considered statistically significant CAIT: Cumberland Ankle Instability Tool; NPRS: Numerical Pain Rating Scale; FAAM: Foot and Ankle Ability Measure

Outcome	Groups	Pre	Post	T value	p value
CAIT	Group A	16.79 ± 2.47	28.10 ± 1.21	22.44	<0.0001
	Group B	16.38 ± 2.76	25.83 ± 0.85	20.70	<0.05
NPRS	Group A	7.66 ± 0.81	1.66 ± 0.55	45.69	<0.0001
	Group B	7.59 ± 0.68	3.34 ± 0.72	33.13	<0.05
FAAM	Group A	45.41 ± 3.52	74.28 ± 3.76	27.76	<0.0001
	Group B	46.45 ± 4.02	58.00 ± 3.71	12.65	<0.05

The postintervention comparison between Groups A and B revealed statistically significant differences across all three outcome measures (p < 0.0001), confirming that Group A achieved superior results compared with Group B. Findings collectively indicate that the intervention administered to Group A was significantly more effective in improving ankle stability, reducing pain, and enhancing overall functional ability, as mentioned in Table 5.

**Table 5:** Comparison of posttest scores of outcomes of Groups A and B Between-group postintervention differences were analyzed using the independent-samples t-test; a p value of <0.05 was considered statistically significant CAIT: Cumberland Ankle Instability Tool; NPRS: Numerical Pain Rating Scale; FAAM: Foot and Ankle Ability Measure

Outcomes	Groups	Post	T value	p value
CAIT	Group A	28.10 ± 1.21	9.982	<0.0001
	Group B	25.83 ± 0.85		
NPRS	Group A	1.66 ± 0.55	10.494	<0.0001
	Group B	3.34 ± 0.72		
FAAM	Group A	74.28 ± 3.76	9.21	<0.0001
	Group B	58.00 ± 3.71		

## Discussion

The present study aimed to evaluate and compare the impact of RST and Conventional Strengthening on ankle joint stability, pain levels, and functional walking ability among individuals presenting with CAI. The results revealed that both treatment approaches led to meaningful improvements across all three measured outcomes: CAIT scores, NPRS scores, and FAAM scores over the four-week intervention period. However, the RST group showed comparatively greater improvements in joint stability and walking performance, while both groups demonstrated comparable reductions in pain intensity.

The significant improvement in CAIT scores observed in the RST group is broadly consistent with the work of Yin et al., whose systematic review and meta-analysis on PNF in CAI patients found improvements in joint position sense and dorsiflexion range of motion following PNF interventions; however, the authors noted these improvements were not significantly different from control groups in their pooled analysis, and called for further research to confirm PNF's specific contribution [10]. The present study's RST group showed a clearer between-group advantage than the meta-analysis demonstrated, suggesting that RST delivered as a distinct, structured protocol rather than as a component within heterogeneous PNF interventions may yield more consistent gains.

Furthermore, Han et al. performed a systematic review and network meta-analysis examining the effect of therapeutic exercises on proprioception in CAI, confirming that neuromuscular training approaches targeting co-contraction and joint position sense yielded the greatest improvements in proprioceptive outcomes, thereby supporting the superior joint stability gains observed with the RST in the present study [17].

Bello et al. reported in a small controlled clinical trial that rhythmic stabilization was associated with fewer or less severe lower limb injuries than conventional passive stretching in indoor soccer athletes, further reinforcing the present study's finding that RST confers greater functional benefits than conventional approaches in populations requiring dynamic ankle stability [18].

The reduction in NPRS scores observed in both groups following the four-week intervention is reinforced by evidence from Yekdaneh and Mutlu, who, in a randomized controlled study investigating proprioception improvements through balance training in individuals with CAI, reported significant reductions in pain alongside improvements in proprioceptive and functional outcomes. The comparable pain reduction observed in both groups in the existing study suggests that any structured exercise intervention, whether neuromuscular or strength-based, is effective in reducing pain associated with CAI, likely through mechanisms including reduced mechanical joint stress, improved muscular support, and modulation of nociceptive pathways via progressive loading [19].

The superior improvements in FAAM scores observed in the RST group align with the findings of Feger et al., who demonstrated that in people with CAI, gait training resulted in notable improvements in neuromechanical outcomes during walking, including enhanced muscle activation patterns, improved ground reaction force distribution, and more symmetrical gait kinematics [20].

These neuromechanical improvements are closely paralleled by the functional walking performance gains reflected in the FAAM scores of Group A in the present study. Additionally, Elabd et al. reported that CAI individuals exhibit significantly altered gait loading strategies compared with healthy controls, and that targeted rehabilitation addressing neuromuscular deficits can meaningfully restore normal loading patterns during ambulation, further corroborating the walking performance improvements observed following the RST protocol [7].

The findings of the present study are well supported by evidence from multiple peer-reviewed, high-impact, indexed publications, strengthening the credibility and clinical relevance of the results. The methodological rigor of the present study, incorporating validated outcome measures, a randomized comparative design, and a structured, progressive four-week protocol, aligns with standards upheld in the existing literature. Together, these results support incorporating RST as a clinically superior, evidence-based intervention into standard physiotherapy protocols for CAI.

Clinical implications

The results of the present study provide direct clinical evidence supporting the incorporation of RST as a superior intervention over conventional strengthening for improving joint stability and walking performance in people with CAI. Physiotherapists managing CAI can adopt the progressive four-week RST protocol as a structured, evidence-based rehabilitation approach that simultaneously addresses neuromuscular deficits, proprioceptive impairments, and functional walking demands. The comparable pain reduction observed suggests that any structured exercise intervention is beneficial; however, RST offers additional neuromuscular advantages that conventional strengthening alone cannot provide.

Strengths

The current analysis shows a number of noteworthy methodological strengths, including a comparative randomized design with an adequate sample size of 58 participants, which provides sufficient statistical power to detect meaningful between-group differences. The use of three validated, reliable, and disease-specific outcome measures, CAIT, NPRS, and FAAM, ensured comprehensive assessment across all relevant domains of joint stability, pain, and functional walking performance. The structured, progressive four-week protocol, with clearly defined weekly progressions for both groups, further enhances the reproducibility and clinical applicability of the study findings.

Limitations

The present study was limited by the absence of a long-term follow-up assessment beyond the four-week intervention period, making it difficult to determine the durability of the observed improvements over time. The study was conducted at a single location, which may limit the generalizability of the results to broader, more diverse clinical populations. Additionally, the absence of blinding of participants and treating therapists because of treatment strategies may have introduced performance bias, and objective biomechanical or electromyographic measures were not incorporated to supplement the self-reported outcome data. The study did not specify which muscles were targeted by the interventions. The ligamentous structures involved were those assessed via the Anterior Drawer Test and Talar Tilt Test (anterior talofibular and calcaneofibular ligaments). The lack of specific instrumental tests, such as isokinetic dynamometry or electromyography, is also a limitation.

Future scope

Future scope for the present study is to evaluate the sustainability of gains in joint stability, pain, and walking performance after both therapies; future research should include longer follow-up periods of three to six months. The external validity of these results would be improved by multicenter randomized controlled trials with larger and more diverse sample populations, including older adults and athletes. Furthermore, future studies that combine patient-reported outcomes with objective biomechanical assessment instruments such as force platforms, electromyography, and isokinetic dynamometry would provide a more comprehensive view of the neuromuscular mechanisms underlying the superior efficacy of RST in CAI rehabilitation.

## Conclusions

The present study found that both RST and Conventional Strengthening are effective interventions for improving joint stability, reducing pain, and enhancing walking performance in individuals with CAI over a four-week intervention period. However, the RST demonstrated comparatively superior improvements in joint stability as measured by the CAIT and walking performance as measured by the FAAM. Therefore, it is concluded that RST is a clinically superior, evidence-based rehabilitation intervention and its integration into standard physiotherapy protocols for CAI is strongly recommended.
